# Molecular Impact of Metabolic and Endocrine Disturbance on Endometrial Function in Polycystic Ovary Syndrome

**DOI:** 10.3390/ijms26209926

**Published:** 2025-10-12

**Authors:** Jim Parker, Claire O’Brien, Talat Uppal, Kelton Tremellen

**Affiliations:** 1 School of Medicine, University of Wollongong, Wollongong 2522, Australia; 2 Faculty of Science and Technology, University of Canberra, Canberra 2617, Australia; claire.obrien@canberra.edu.au; 3 Faculty of Medicine and Health Sciences, Macquarie University, Macquarie Park, NSW 2109, Australia; drtalat@womenshealthroad.com.au; 4 Department of Obstetrics and Gynaecology and Reproductive Medicine, Flinders University, Bedford Park, SA 5042, Australia; kelton.tremellen@flinders.edu.au

**Keywords:** polycystic ovary syndrome, endometrium, metabolic, hormonal, endocrine, dysfunctional uterine bleeding, pregnancy, decidualization, postmenopausal, cancer

## Abstract

Polycystic ovary syndrome (PCOS) is a systemic metabolic and endocrine disorder that significantly disrupts reproductive physiology and endometrial function. In this narrative review, we examine the molecular impact of metabolic and hormonal imbalances on the endometrium of women with PCOS. We investigate the specific mechanisms that delineate how hyperinsulinemia and insulin resistance, chronic low-grade inflammation, and estrogen/progesterone/androgen imbalance contribute to altered epigenetic, transcriptomic, metabolomic, and signaling profiles in a wide array of different cell types within endometrial tissues. The synergistic interplay between upregulated inflammatory cytokines (e.g., IL-1,2,6,8,17,18, and TNF-α), along with key changes in critical molecular pathways associated with hyperinsulinemia and insulin resistance (e.g., PI3K/AKT/MAPK, and Wnt/β-catenin), in addition to aberrant sex steroid hormone signaling (e.g., CYP19A1, COX-2, PGE_2_, HOXA10, 11βHSD2), promotes deleterious changes within the endometrial microenvironment. These anomalies underpin a spectrum of clinical manifestations observed in women with PCOS at each stage of the life course, including abnormal uterine bleeding in reproductive-age women, impaired decidualization in pregnancy, and altered postmenopausal endometrial physiology. Clinically, these alterations are associated with abnormal uterine bleeding, subfertility, implantation failure, miscarriage, pregnancy complications, and postmenopausal endometrial hyperplasia and cancer. Overall, our review provides novel insights into the molecular mechanisms linking systemic metabolic and endocrine dysfunction with endometrial pathology in PCOS and has broader implications that apply to all women.

## 1. Introduction

Polycystic ovary syndrome (PCOS) is increasingly being viewed as an evolutionary mismatch disorder that becomes apparent after exposure to contemporary lifestyle, nutritional, and environmental factors [1,2,3]. Having a metabolically thrifty metabolism was an evolutionary advantage in times of famine, as it enhanced the capacity to store energy (e.g., triglycerides) for later use in times of food scarcity [1]. However, in today’s environment, characterized by diminished diet quality, calorie surplus, sedentary behavior, and pervasive lifestyle shifts, these previously advantageous phenotypes have become maladaptive, exerting detrimental effects on both metabolic and reproductive health. Consistent with the evolutionary models, the international guidelines on PCOS provide evidence that many of its symptoms and biochemical and endocrine changes are reversible following diet, exercise, and other lifestyle and medical interventions [4]. It is anticipated that characterizing PCOS from an evolutionary perspective may help create a framework conducive to promoting a healthy lifestyle and preventative interventions [5].

PCOS affects 8–13% of women globally and encompasses a constellation of metabolic and endocrine disturbances that define it as a true systemic disorder [6,7]. This is reflected in the current international consensus process to change the name to reflect the contribution of metabolic and endocrine factors [4,8]. Analysis of the Global Burden of Disease Study’s data found that the global prevalence of PCOS and related infertility is increasing [9]. The escalating prevalence of PCOS in lower socio-demographic regions is particularly concerning. The authors identified environmental and behavioral risks—such as high-fat/high-sugar diets, sedentary behavior, stress, circadian disruption, and exposure to endocrine-disrupting chemicals (EDCs)—as the fundamental drivers [9].

Key features of PCOS, including insulin resistance (IR), hyperinsulinemia, low-grade chronic systemic inflammation (CSI), and hyperandrogenism (HA), act synergistically to impair endometrial function, leading to menstrual disturbance, reduced fertility, and adverse obstetric outcomes [10,11,12]. Although PCOS is now considered a multisystem disorder, the precise molecular pathways that induce endometrial dysfunction are still under investigation [11,13,14]. The convergence of high-throughput profiling (e.g., transcriptomics), advanced bioinformatics (Gene Set Enrichment Analysis and Connectivity Map), and novel in vitro models (e.g., organoids) is providing insights into how molecular networks regulate endometrial function [15,16,17].

Emerging evidence implicates altered insulin receptor signaling (phosphoinositide 3-kinase/serine-threonine-specific kinase–mitogen-activated protein kinase (PI3K/AKT/MAPK) [18], androgen-mediated shifts in Wilms tumor-1 (WT1) (a transcription factor important for cell development and survival [19]), and Wingless-related integration site (Wnt)/β-catenin activity [20] in disrupting precisely coordinated hormone-induced changes in the PCOS endometrium [21,22]. At the same time, elevated inflammatory cytokines, such as tumor necrosis factor alpha (TNF-α) and interleukin-6 (IL-6), disrupt local hormone networks by interfering with estrogen (E2), progesterone (P4), and insulin receptor signaling [11,23]. Together, the resulting signaling defects alter epigenetic regulatory pathways [24], change the endometrial transcriptome (the set of all RNA transcripts) [25] and metabolome (the collection of small molecules produced by a cell) [26], and impair cell-to-cell communication by disrupting gap junction and paracrine signaling networks [14]. Ultimately, the disruption of finely tuned proliferative and secretory phase hormonal and metabolic signaling networks plays a pivotal role in the pathophysiology of endometrial dysfunction.

Clinically, these molecular events have unique effects at different life stages. In reproductive-age women, they may present as altered bleeding patterns, excessive endometrial proliferation, impaired fertility, and reduced implantation potential [27]. In pregnancy, altered bidirectional communication leads to impaired stromal decidualization and trophoblast invasion, increasing the risk of miscarriage, preterm birth, preeclampsia, and other pregnancy complications [28]. After the menopausal transition, residual metabolic, inflammatory, and hormonal dysregulation may promote endometrial overgrowth and enhance neoplastic potential [29].

This narrative review integrates advances in our understanding of how IR, CSI, and E2/P4/androgen imbalance remodel the endometrial environment at a molecular level. Our aim is to highlight the role of the core pathophysiological components of PCOS in endometrial dysfunction. This should inform future research and guide precision-based approaches to the prevention and management of endometrial health across the lifespan.

## 2. Scope and Methodology

Section 3—Endometrial Function in Reproductive-Age Women—is a summary of normal endometrial function in women of reproductive age and provides the background for subsequent sections on the effect of IR, hyperinsulinemia, CSI, and hormonal imbalance on normal physiology. The normal role of the mucosal immune system and related microbiome (MB) is discussed. Readers are directed to the corresponding discussion in Section 4 to link normal endometrial physiology with the currently available mechanistic descriptions of the pathophysiology.

Section 4—Pathophysiology of Dysfunctional Endometrium—provides a review of endometrial changes related to IR, CSI, and E2/P4/androgen imbalance, particularly in women with PCOS. This section also includes a review of the molecular alterations in abnormal uterine bleeding (AUB) and heavy menstrual bleeding (HMB). This section provides an overview of the place of PCOS in the classification of AUB, followed by a detailed molecular description of endometrial events in AUB and PCOS. Section 4 also includes a summary of emerging research from endometrial organoid models.

Section 5 is a discussion of the implications of the novel findings identified in this review, gaps in molecular understanding, and recommendations for future research. Section 5.1—*Mechanistic Implications of the Novel Findings Identified in this Review*—expands on novel insights into endometrial dysfunction identified in Section 4. This includes molecular changes in PCOS and the broader relevance of these findings to female reproductive health. In Section 5.2—*Current Gaps in the Molecular Understanding of Endometrial Dysfunction in Polycystic Ovary Syndrome*—we identify and summarize critical gaps in our current understanding of key pathways, cell function, multiomics, and descriptive associations of endometrial dysfunction. In Section 5.3—*Development of Precision Research and Targeted Therapeutic Strategies*—we provide explicit examples of how this review could inform future research.

Section 6—Limitations of the Current Narrative Review—outlines the limitations of the narrative review format with respect to inclusion criteria, study quality, and translation of the findings into clinical practice.

Our list of bibliographic references is based on MEDLINE, PubMed, Scopus, Google Scholar, and Cochrane databases. Databases were searched from inception to September 2025. Papers reviewed include primary research, narrative reviews, and systematic reviews. Additional articles were obtained from the bibliography list of retrieved publications. To optimize our literature search, we also employed Microsoft Copilot (GPT-4; Microsoft 2025) to locate relevant articles using keywords and phrases. The resulting narrative synthesis aims to provide a contemporary summary of molecular research on endometrial dysfunction in PCOS and women with AUB.

We synthesized the data qualitatively, and no attempt was made to perform a systematic review due to a number of methodological and scientific reasons. The broad interdisciplinary scope of the molecular impact of IR, CSI, and hormonal disturbance on the endometrium spans epigenetics, transcriptomics, metabolomics, various experimental models (e.g., single cell, organoids, animal, and human), clinical phenotypes, and nutritional and environmental exposures. This review covers a heterogeneous evidence base that includes studies, from single-cell RNA sequencing and chromatin immunoprecipitation experiments to organoid culture and epidemiological and randomized lifestyle interventions. The diversity in models, endpoints, and outcome measures precludes uniform inclusion and exclusion criteria, risk-of-bias assessments, and quantitative pooling of data, as is required for a systematic review.

## 3. Endometrial Function in Reproductive-Age Women

**Figure 1:**
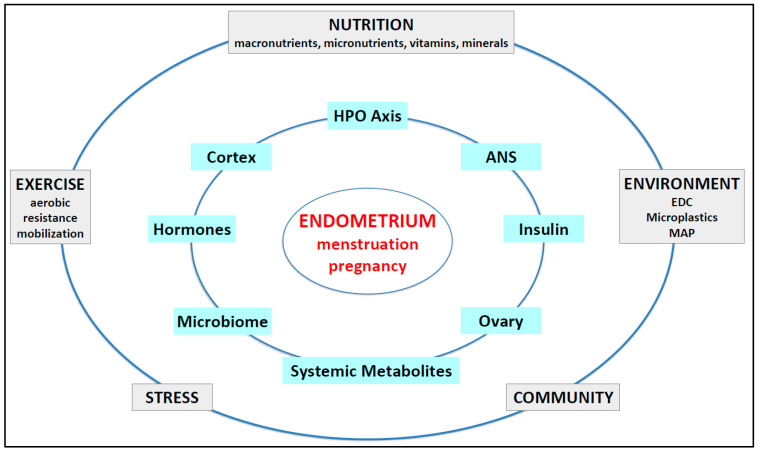
Spheres of influence that determine menstrual function in polycystic ovary syndrome. grey = nutritional, environmental and lifestyle causes; blue = systemic causes; red = end organ responses. Abbreviations: HPO = hypothalamic pituitary–ovarian; ANS = autonomic nervous system; EDC = endocrine-disrupting chemical; MAP = microparticulate air pollution.

The human endometrium is the functional tissue that interacts with the embryo from the next generation to ensure implantation, appropriate feto-placental development and growth, and species survival [10,30,31]. Female metabolism and endocrine physiology are intimately connected by reciprocal feedback mechanisms that ensure reproduction is coordinated with optimal metabolic health [1,3]. Cyclical stimulation of endometrial epithelial, stromal, glandular, and muscular tissues by ovarian-derived sex steroids and locally produced paracrine hormones, cytokines, chemokines, exosomes, and other communication molecules operates best when female physiology and metabolism are optimized. Lifestyle factors such as a healthy diet and exercise are recommended for first-line treatment of PCOS in the international guidelines to optimize physiology; metabolism; and, ultimately, molecular function in the endometrium (see Figure 1).

Altered physiology in the hypothalamic–pituitary–ovarian (HPO) tissues can downregulate and limit reproduction in times of metabolic and systemic stress [1,2]. Pathophysiological changes, such as IR, CSI, HA, and other hormonal disturbances, can impact the endometrium indirectly via effects in distant tissues (ovaries, adipose, liver, pancreas, and brain) or cause direct negative effects in endometrial cells and tissues [21]. This can result in endometrial changes that are reflected in multiple symptoms and pathologies that vary across the lifespan (see Section 4) [32].

### 3.1. Normal Endometrial Physiology

Cyclical menstruation is a sign of both reproductive and metabolic health. The American College of Obstetricians and Gynecologists and the American Academy of Pediatrics have recommended using the menstrual cycle as a vital sign [33,34]. Abnormalities in cycle length or volume can both signal and exacerbate chronic conditions, making cycle tracking a valuable assessment tool from adolescence through menopause [35,36]. Furthermore, elevated insulin levels, low-grade inflammatory mediators, and altered hormones appear to crosstalk with HPO signals and endometrial responses, undermining normal cyclical menstrual function (see Section 4.3).

### 3.2. Regulation of Hypothalamic–Pituitary Gonadotrophin Hormones

Ovarian function is driven by three-way regulatory feedback circuits linking the hypothalamus, pituitary, and ovaries [37]. Studies of cell cultures, tissue cultures, and animal models show that hypothalamic neurons release a 10-amino-acid hormone, gonadotropin-releasing hormone (GnRH), under the control of arcuate–nucleus neurons co-expressing kisspeptin, neurokinin B, and dynorphin (the KNDy network) [37,38]. Those KNDy cells generate GnRH in discrete bursts into the hypophyseal portal blood vessels, prompting the anterior pituitary to discharge follicle-stimulating hormone (FSH) and luteinizing hormone (LH) [38]. Slow GnRH oscillations (about one burst every 2–3 h) bias the pituitary toward FSH output, while accelerated rhythms (one burst every 30–60 min), as is often observed in PCOS, drive up LH release and increase the LH–FSH ratio [39].

The pulsatile activity of GnRH is mainly regulated by indirect homeostatic feedback from gonadal steroid hormones to the neuronal network upstream of GnRH neurons [39]. Specifically, GnRH neurons themselves carry estrogen receptor β (ERβ), while ERα, progesterone receptor (PR), and androgen receptor (AR) are localized to upstream KNDy and gamma-aminobutyric acid circuits [40]. When ovarian steroid signals are perturbed—whether by metabolic derangements, inflammation, or shifts in hormone levels—those neural circuits lose their ability to synchronize GnRH release, undermining normal HPO-axis regulation of ovarian hormone production and folliculogenesis.

### 3.3. Hormonal Control of Ovarian Hormones—Indirect Control of the Endometrium

#### 3.3.1. Sex Steroid Regulation of the Endometrium—Systemic Endocrine Control

The two-cell/two-gonadotropin interplay between theca and granulosa cells ensures precise control of androgen production by LH and its conversion into estrogens under FSH influence, fine-tuning hormone output for folliculogenesis and endometrial cycling [41]. Ultimately, ovarian hormones enter the systemic circulation and exert specific molecular actions on endometrial cells. E2-ER signaling upregulates paracrine growth factors such as cyclin proteins, which regulate cell division [42], and insulin-like growth factor-1 (IGF-1) [43], which drives cell proliferation during the follicular phase [44,45]. In addition, E2 signaling increases vascular endothelial growth factor (VEGF) and angiogenesis [46,47] and alters glandular epithelium and stromal cell function [48].

Following ovulation, P4-PR signaling induces secretory changes in the endometrium, followed by decidualization in stromal cells around cycle day 23 (e.g., FOXO1, IGF-binding protein-1, and prolactin) [49]. P4-PR signaling also suppresses transforming growth factor β-mediated increases in matrix metalloproteinases (MMPs) [50] and proinflammatory cytokines [47]. In the absence of pregnancy, P4 withdrawal activates nuclear factor kappa-light-chain-enhancer of activated B cells (NFκB), which elevates inflammatory cytokines (IL-1β, TNF-α) and MMPs, inducing spiral arteriolar vasoconstriction and focal hypoxia in the functional layer, triggering endometrial tissue breakdown, repair, and regeneration. Disruption to sequential sex steroid production in the ovaries from adverse systemic factors, such as IR and CSI, makes a significant contribution to endometrial dysfunction (see Section 4.3.1).

#### 3.3.2. Intracrine Metabolism in the Endometrium—Local Hormonal Control

Beyond the influence of circulating hormones, the endometrium orchestrates its own steroid environment by expressing local intracrine enzymes [51,52]. These tissue-specific steroidogenic and metabolic enzymes convert inactive precursors—like dehydroepiandrosterone (DHEA) and DHEA-sulfate—into bioactive sex steroids within selected endometrial cell populations, providing the endometrium with precise, site-specific control over hormone-driven processes [53].

Advances in liquid chromatography–tandem mass spectrometry have allowed for more accurate profiling of intracrine sex steroids [54]. Endometrial tissue levels of E2 can be 5–8 times higher than those of serum during the proliferative phase and half the concentration in the secretory phase [55]. Levels of P4 are similar in tissue and serum, suggesting that local levels are determined by passive diffusion [56]. Testosterone levels are lower in endometrial tissue than in serum and do not show cyclical variation [56]. This may reflect the dual role of androgens as ligands for AR and substrates for E2 biosynthesis. Nevertheless, AR signaling has been found to regulate genes involved in cytoskeletal organization, cell motility, and cell cycle progression [57]. Testosterone also inhibits the production of MMP-1 in cultured human endometrial stromal cells in a similar manner to P4 [58]. MMP-1 is involved in the regulation of menstruation [59] and implantation [60]; therefore, elevated androgens in PCOS may have pathophysiological significance (see Section 4.3.2).

### 3.4. The Endometrium as a Component of the Mucosal Immune System

The mucosal immune system and the human MB form an interconnected network that spans the entire body [61]. Each mucosal interface has its own unique MB—which refers to the entire habitat of microorganisms, including the genome and surrounding environmental conditions—and microbiota (collection of microorganisms) [62]. The mucosal immune system and MB act as an adaptive interface between the host and the environment, constantly recalibrating to maintain health [63]. Disruptions in this symbiosis, through diet, environmental influences (e.g., EDCs, microparticulate air pollution, or microplastics), infection, or antibiotics, can trigger mucosal immune dysregulation and contribute to a range of local and systemic conditions, such as infertility, pregnancy complications, and PCOS [10,64,65,66]. In addition, disturbances in the MB at one mucosal site can contribute to IR, CSI, and hormonal imbalance, which have pathological influences in apparently unrelated tissues and organs, such as the ovaries and endometrium (see Section 4.8).

The endometrial mucosal immune system and its resident MB form a dynamic, interdependent ecosystem crucial for normal menstrual function and reproductive success [67]. The endometrium is lined by a single layer of columnar epithelial cells that is joined by tight junctions and covered by mucous, which forms a selective barrier that prevents pathogen entry while permitting nutrient and hormone exchange [68]. The endometrial mucosal-associated immune tissue consists of numerous effector cells from the innate and adaptive immune systems. Together, endometrial epithelial cells and mucosal leukocytes produce antimicrobial proteins that vary throughout the menstrual cycle and pregnancy (B-defensins, secretory protease inhibitors, and immunoglobulins) [69]. In addition to its protective function, the endometrial immune system has a unique role in enabling fertilization, implantation, placental function, pregnancy, and menstruation [68].

E2 and P4 tightly regulate endometrial immunity and immune–stroma crosstalk [70]. E2 enhances epithelial barrier integrity, modulates cytokine production, and promotes inflammation (e.g., leukemia inhibitory factor). P4, acting via P4 and the glucocorticoid receptor, dampens proinflammatory signals during the secretory phase of the menstrual cycle, in part via inhibition of NFκB. Immune cells (T lymphocytes, natural killer cells, and macrophages) engage in bidirectional crosstalk with stromal and epithelial cells, ensuring immunological tolerance to semen and embryos while maintaining defense against pathogens [68]. Dysregulated sex steroid signals and adverse systemic responses—such as IR, CSI, and E2/P4/androgen imbalance in PCOS—can impair mucosal immune defenses, altering cytokine secretion and immune cell recruitment in the endometrium, thereby contributing to reproductive and menstrual disturbances (see Section 4.5) [71].

### 3.5. Endometrial Inflammation and Leukocyte Function

Menstruation represents a cascade of inflammatory events orchestrated by hormone withdrawal, immune cell recruitment, and the release of inflammatory mediators. P4 withdrawal releases the inhibition of NFκB in decidualized stromal cells, promoting transcription of proinflammatory genes [72]. This initiates inflammasome formation—which involves the assembly of the nod-like receptor family pyrin domain containing 3-apoptosis-associated speck-like protein-caspase-1 complex—leading to the release of chemokines IL-1B and IL-18, which promote leukocyte influx into the endometrium. Recruited immune cells secrete MMPs that break down the extracellular matrix and enable tissue breakdown at the onset of menstrual bleeding [73].

The inflammatory process appears to be self-limited in physiological menstruation. For example, IL-1 (pro-inflammatory) increases the expression of 11βHSD1, which converts cortisone into cortisol (anti-inflammatory). Glucocorticoids downregulate inflammation by increasing transcription of anti-inflammatory genes, decreasing pro-inflammatory transcription factors, and limiting production of cytokines [74]. In addition, P4 withdrawal promotes phospholipase A2 activity, releasing arachidonic acid from endometrial cell membranes, which is converted by cyclooxygenase-2 into prostaglandins (PGs), which drive myometrial contractions and spiral arteriolar vasoconstriction [75]. Low-grade CSI can have direct and indirect effects on the endometrium, and it contributes to endometrial dysfunction and AUB in women with PCOS (see Section 4.4).

### 3.6. Role of Vasoconstriction, Hypoxia, and Hemostasis in Menstruation

Menstrual bleeding commences after a tightly coordinated series of events that constrict spiral arterioles to limit bleeding, followed by repair signals generated by short-lived low oxygen levels, and finally from activation of the coagulation cascade to seal ruptured vessels [76]. Early in menses, local vasoconstrictors—such as PGF_2_α and endothelin-1—cause the transient vasoconstriction of spiral arterioles in the functional layer [76]. The resulting local hypoxia prevents the degradation of hypoxia-inducible factor-1α, which upregulates genes for new vessel growth and tissue regeneration, such as VEGF, and chemokines, such as chemokine receptor type 4 [77]. As vessels rupture, decidual stromal cells express high levels of tissue factor, triggering the extrinsic clotting pathway [78]. Platelets adhere to exposed collagen, forming a fibrin matrix, while thrombin stops bleeding and facilitates extracellular matrix remodeling, which is essential for repair [79]. Disruption at any point can manifest as dysmenorrhea, HMB, or delayed endometrial repair (see Section 4.6).

### 3.7. Endometrial Stem Cells

Over the reproductive lifetime, the uterine lining renews itself hundreds of times through a monthly cycle of shedding and restoration. This repeated turnover depends on stem and progenitor cells residing within the basal stratum, which survive the menstrual breakdown and regenerate the functional compartment [80]. In the first two days after menstruation, these precursors rapidly expand to reform the epithelial surface [81]. During the proliferative phase, E2 drives their proliferation and orchestrates scar-free rebuilding of the endometrium. These stem cells also secrete growth factors and signaling molecules—such as VEGF and cysteine-rich angiogenesis inducer 61—which promote new vessel growth, fine-tune immune cell trafficking, and support stromal expansion along with matrix remodeling [82]. Disruption of this communication network impairs decidualization and predisposes people to reproductive disorders and AUB (see Section 4.7).

Dysfunction of endometrial stem cells can underlie disorders such as Asherman’s syndrome (impaired repair) and HMB (excessive breakdown), and stem cell therapy may have a future role in the treatment of PCOS [83]. Stem cell therapy has been investigated in a number of reproductive disorders. In PCOS, the majority of studies have examined the impact of stem cell treatment on ovarian function [84]. Mesenchymal stem cell therapy has been found to improve endocrine [85] and metabolic function (via the PI3K/AKT and FOXO signaling pathway) [86], enhance oocyte quality [87], and reduce the synthesis of proinflammatory cytokines (IL-1β, IL-10, TNF-α, and interferon-γ) [88] in cell and animal models. Human endometrial stem cells can also be used to develop organoid systems that mimic native endometrial tissue and permit detailed molecular investigation of endometrial physiology and dysfunction [89] (see Section 4.9).

## 4. Pathophysiology of Dysfunctional Endometrium

### 4.1. Classification of Abnormal Uterine Bleeding (AUB) and Heavy Menstrual Bleeding (HMB)

The etiology of AUB is categorized into structural and non-structural causes using the International Federation of Gynecology and Obstetrics (FIGO) PALM-COEIN system [90]. Approximately one-third of women are affected by AUB, which can have a significant impact on quality of life [91]. HMB occurs in up to 25% of women and is the most common form of AUB [92]. HMB is defined objectively by having measured menstrual blood loss of >80 mL [93]. However, in a clinical setting, HMB is usually defined subjectively using visual methods and is reported in up to 50% of women [93]. This aligns with the more person-centered definition of HMB as “excessive menstrual blood loss that interferes with a woman’s physical, social, emotional, and/or material quality of life” outlined by FIGO [90], and adopted by the National Institute for Health and Care Excellence guidelines [94] and by the Australian Commission on Safety and Quality in Health Care Clinical Standards [95].

HMB can be associated with oligomenorrhea, iron-deficiency (present in 60% of women at presentation), iron deficiency anemia—which occurs in one in four people with HMB—lost productivity from work (estimated at 30 days per year) [96], decreased physical activity and sexual function, and reduced overall quality of life, and it is a substantial healthcare burden [97]. Although most women with PCOS have AUB (70–80%), there is a scarcity of data on the prevalence of HMB in this patient group. A cross-sectional study from China examining the relationship between pubertal timing and menstrual characteristics did not show an association between PCOS and HMB, although the majority of women with PCOS had regular menstrual cycles and normal BMI [98]. The management of HMB by General Practitioners is increasing [99], and a large longitudinal cohort survey found that women with PCOS were more likely to experience HMB [100]. In addition, HMB was found to increase with age (17.6% at age 22 years to 32.1% at 48 years) and body mass index (BMI). The increased prevalence of HMB in women with elevated BMI and in PCOS suggests a role for modifiable risk factors in the pathogenesis and management of HMB.

Elevated BMI is also associated with an increased risk of structural causes of HMB, including uterine polyps [101] and fibroids [102]. A recent systematic review of 33 articles reported that high BMI was the most significant risk factor for fibroids [102]. Healthy nutrition (high fruit and vegetable intake), high sun exposure, and increased vitamin D intake were protective for uterine fibroid development. In addition, a large phenome-wide association study revealed that women with fibroids had a statistically significant increase in multiple coexisting chronic diseases, suggesting shared pathophysiological processes [103]. Given that overweight and obesity are often associated with similar underlying metabolic dysfunction to that found in PCOS, such as IR, hyperinsulinemia, CSI, and hormone imbalance, there may be shared molecular pathways that contribute to HMB in both structural and non-structural causes of AUB.

Importantly, nearly all metabolic and endocrine features related to PCOS also influence endometrial function, independent of PCOS [104]. As a result, there is a significant overlap in the observed molecular changes in the endometrium of women with PCOS and unexplained AUB and HMB (e.g., IL, TNFα, MMP, PG, and VEGF) (see Section 4.2, Section 4.3, Section 4.4, Section 4.5, Section 4.6, Section 4.7 and Section 4.8). These include molecular changes in the endometrium in the PALM-COEIN non-structural categories of AUB-Ovulatory dysfunction type-4 (PCOS) and AUB-Endometrial (E). AUB-E is a diagnosis of exclusion after structural lesions (e.g., polyps, fibroids, adenomyosis, and cancer), coagulopathy (e.g., von Willebrand disease), and not otherwise classified causes (e.g., arteriovenous malformation and isthmocele) have been ruled out [90]. Therefore, there may be common modifiable risk factors that contribute to many seemingly unrelated causes of AUB and HMB.

The uterine endometrium is an end organ and can be impacted by direct and indirect factors. Preexisting factors, like IR, elevated insulin levels, and CSI, can impair ovarian aromatase activity and skew the E2–androgen ratio [105]. Such an imbalance may have an indirect impact on the endometrium by disrupting follicle development, inhibiting ovulation and corpus luteum formation, and ultimately blocking the P4 surge required for endometrial decidualization during the secretory phase of the menstrual cycle. In addition, IR, CSI, and hormone imbalances can have direct effects on signaling networks in endometrial cells, and this is discussed in the following sections [106]. There is a range of clinical problems related to abnormal endometrial function in PCOS (Table 1).

### 4.2. Association Between IR, Hyperinsulinemia, and Menstrual Dysfunction

Previous studies have demonstrated a relationship between irregular menstrual cycles and the severity of IR in women with [128,129,130] and without PCOS [131,132]. As a result, the degree of menstrual dysfunction has been proposed as a surrogate marker of IR in PCOS [128,132]. Other factors—such as elevated body weight and HA—may also exacerbate both the degree of IR and menstrual disturbance [129].

Menstrual disturbance has also been reported to be a risk factor for type 2 diabetes mellitus (T2DM), which is characterized by IR plus elevated blood glucose levels. Although not all studies report an elevated risk [133], many studies—including several large-scale longitudinal cohorts—have demonstrated an association [131,134]. The Nurses’ Health Study II—a large prospective cohort study with 1,639,485 person-years of follow-up—reported a greater risk of T2DM in women with long or irregular menstrual cycles, with greater risk in those with unhealthy lifestyles [134]. The study showed an additive effect of excess weight, inactivity, poor-quality diet, and menstrual cycle dysfunction on the risk of T2DM. The authors emphasized the need to consider menstrual cycle disturbance as an independent predictor of metabolic risk, aligning with recommendations from professional colleges and other reports [33,34,135].

### 4.3. Impact of IR and Hyperinsulinemia on Endometrial Dysfunction

It is well established that metabolic risk factors—such as IR, obesity, and metabolic syndrome—are associated with structural causes of AUB, such as polyps [136,137] and leiomyoma [138,139]. Similarly, non-structural causes of AUB—such as AUB-O type 4 (PCOS) and AUB-E—are also associated with metabolic risk factors, such as IR, CSI, and obesity, via both indirect and direct mechanisms (see Section 4.2). It is estimated that almost half of all individuals with HMB referred for secondary care do not have an identifiable cause [140]. Preventable and reversible metabolic risk factors may therefore contribute to the majority of cases of AUB, including structural and non-structural (e.g., PCOS and unexplained HMB).

#### 4.3.1. Indirect Effect of IR and Hyperinsulinemia on Endometrial Dysfunction via Altered Ovarian Hormone Production

Hyperinsulinemia and IR significantly disrupt ovarian function via a number of local and systemic mechanisms. Insulin increases GnRH pulse frequency and LH release and enhances theca cell responsiveness to LH via the PI3K, MAPK, and inositolglycan signaling systems, enhancing ovarian androgen production [141,142,143,144]. IR, hyperinsulinemia, and related defects in post-receptor signaling pathways—such as serine phosphorylation of insulin receptor substrate-1 (IRS-1), AKT phosphorylation, and GLUT4 translocation—have been shown to disturb glucose metabolism and enhance androgen biosynthesis in ovarian theca and granulosa cells [145,146]. Hyperinsulinemia directly inhibits FSH-mediated phosphorylation of AKT in cultured luteinized granulosa cells from women with anovulatory PCOS and causes reduced glucose uptake [145].

Insulin-mediated impairment of FSH also downregulates aromatase expression, contributing to androgen excess and decreased ovarian E2 production [147]. As a result, women with PCOS often have serum E2 levels that are in the low to mid part of the normal range, but the window of action can be prolonged due to P4 resistance or anovulation and lack of P4 antagonism (see Section 4.5) [148]. Furthermore, women with PCOS and IR have elevated advanced glycation end-products in follicular fluid that disrupt granulosa cell steroidogenesis, contribute to mitochondrial oxidative stress, and impair ovulation and oocyte quality [149,150]. Together, IR and hyperinsulinemia alter ovarian steroid production and indirectly contribute to endometrial dysfunction.

#### 4.3.2. Direct Effect of IR and Hyperinsulinemia on Endometrial Dysfunction

IR and hyperinsulinemia promote endometrial inflammation and proliferation [151,152]. At a cellular level, hyperinsulinemia activates or disrupts multiple signaling pathways that regulate metabolism, cell survival, apoptosis, proliferation, and endometrial homeostasis [137]. Differentiating the effects of elevated insulin from those of androgens in the endometrium is complex, as they often co-exist and have a bidirectional relationship in PCOS [6,153]. For example, in the human endometrium, HA alters gene expression related to insulin signaling, glucose metabolism, and GLUT4 transport [154]. Furthermore, in PCOS, elevated inflammatory cytokines, such as TNF, suppress insulin receptor substrate-1 activation and impair glucose uptake in the endometrial stromal cells—an effect that is further amplified by rising levels of insulin and testosterone [23]. Nevertheless, many studies provide evidence of an independent effect of IR and hyperinsulinemia on endometrial cells in PCOS.

Insulin and IGF-1 receptors share common signaling pathways but also regulate distinct cellular processes [155]. In endometrial tissue, PI3K/AKT serves as a key conduit for signals from both insulin and IGF-1 [109]. When insulin engages its receptor, PI3K is activated, which in turn phosphorylates AKT. Activated AKT then enhances the survival and proliferation of endometrial cells while blocking apoptotic pathways [156]. Aberrant regulation of the AKT-NR4A1 cascade caused by hyperinsulinemia in human endometrial stromal cells and PCOS-affected rats has been linked to impaired decidualization in PCOS [157]. In these experiments, insulin treatment markedly reduced the mRNA expression of decidual markers in stromal cells. IGF-1 feeds into the same insulin signaling network, boosting cell growth and anti-apoptotic effects [158]. IGF-1 also plays an important role in decidualization, and hyperinsulinemia—along with unopposed E2 and/or HA—may augment the mitogenic activity of IGF-1 in PCOS [13].

Increased insulin exposure has also been shown to increase the expression of heterogenous paired box6 (PAX6)—a master transcription regulator of proliferation in endometrial epithelial cells—in IR-induced cell cultures [151]. In one study, increased PAX6 repressed the cyclin-dependent kinase inhibitor 1B gene expression of p27 protein—an important negative regulator of endometrial cell cycle progression—in an insulin-dependent manner. In addition, PAX6 mRNA expression was higher in PCOS endometrial tissue than in controls [151]. These findings provide a mechanistic explanation for the link between hyperinsulinemia and endometrial epithelial cell proliferation in PCOS.

In summary, observational, cross-sectional, and retrospective studies support a robust association between menstrual disturbance and IR (Table 2)—which is completely reversible [159,160]. These data are supported by interventional and mechanistic studies showing improved menstrual function following treatment of IR (see Section 4.10) [11,138]. Diet, physical activity, and other lifestyle interventions are the recommended first-line treatment for metabolic dysfunction and IR in PCOS, and additional medical treatment with metformin or inositol supplementation is supported by current evidence [4]. Combination therapy with the oral contraceptive and metformin has been found to improve insulin sensitivity, BMI, the free androgen index, and clinical hyperandrogenism in adolescents with PCOS and hyperinsulinemia [161].

Emerging therapeutic options provide innovations for the prevention and treatment of IR [160]. These include spexin (neuropeptide), sitagliptin (incretin inhibitor), thrombomodulin (coagulation regulator), exenatide, semiglutide, liraglutide (GLP-1 receptor agonists), Tirzepatide (GLP1-/GIP), gliflozins (sodium–glucose cotransporter (SGLT2) inhibitors), clenbuterol (β2-agonist), baicalin (gamma-aminobutyric acid receptor modulator), minerals (magnesium sulfate and calcium), vitamins (K2, D3), nitric oxide donors, and nature-derived compounds (marine oil, flaxseed oil, isoflavones, triterpenes, and anthocyanins). The investigation of these therapeutics has revealed further insight into detailed molecular pathways involved in IR and can be found elsewhere in excellent reviews [11,160].

### 4.4. Chronic Systemic Inflammation (CSI) and Endometrial Dysfunction

Low-grade CSI is a key component in the pathophysiology of PCOS [6,164]. Women with PCOS have higher levels of inflammatory cytokines, including interleukins (IL-1,2,6,8,17,18) and TNF-α, and increased C-reactive protein and leukocytes [164,165]. CSI can have an indirect effect on endometrial function by altering the inflammatory milieu in the ovaries—and ovarian hormone production—or via a direct effect on endometrial cells and tissues [165,166,167]. There is a bidirectional relationship between inflammatory cell imbalances and production of inflammatory cytokines in ovarian and endometrial tissue and the systemic circulation, making it difficult to determine the direction of causality [168]. Nevertheless, the number and composition of immune cell subsets, cytokines, and chemokines in the PCOS endometrium have consistently been found to differ from non-PCOS controls [166,168]. As a result, activation of proinflammatory factors in the endometrium disrupts immune homeostasis, exaggerates normal endometrial inflammation, delays hemostasis and repair, and contributes to HMB [152].

Inflammation plays a significant role in normal menstruation, and PGs perform a number of important regulatory functions in this process (see Section 3.5 and Section 3.6) [72,73,75]. Cyclooxygenase (COX) enzymes—COX-1 and COX-2—convert arachidonic acid into prostaglandins. COX-PG signaling pathways have been found to be upregulated in the endometrium of women with HMB [169]. Gene expression analyses of biopsies from the secretory endometrium of women with measured HMB have shown a significant elevation in COX-1 and COX-2 mRNA compared to women with normal blood loss [169]. PGs mediate their actions via membrane-bound G-protein coupled receptors and increase cAMP after ligand–receptor binding [170]. An in vitro tissue culture of endometrium from women with HMB showed a significant elevation of cAMP after PGE2 stimulation [169]. PG inhibitors have been found to decrease menstrual blood loss by 30–50% [171,172] via inhibition of PG synthesis and decreased PGE2 binding to its receptors [173]. These data suggest that increased endometrial inflammatory changes are important in the pathophysiology of HMB, as described in FIGO AUB-E.

Many factors can contribute to the etiology of CSI in PCOS, including diet [65]; environmental (EDCs, microplastics, and microparticulate air pollution) [174,175]; microbial, gastrointestinal dysbiosis-induced metabolic endotoxemia [64,65]; and other lifestyle factors [176]. These factors can directly modulate CSI, IR, and systemic metabolite levels and are likely to contribute to endometrial dysfunction and HMB [177]. Given that many of these factors are preventable and reversible—and provide increased autonomy and individual control over health—research priority should be given to appropriate intervention trials.

### 4.5. Molecular Changes in the Dysfunctional Endometrium in Ovulatory and Anovulatory PCOS

#### 4.5.1. Endometrial Changes in Ovulatory PCOS

Clinical trials comparing reproductive outcomes in women with PCOS who ovulate with healthy matched controls are not available. Nevertheless, they have a higher rate of implantation failure and reduced pregnancy rates following ovulation induction and in vitro fertilization, suggesting oocyte competence and endometrial dysfunction may play a role [178,179]. Experimental data indicate that ovulatory women with PCOS have alterations in molecular pathways in the ovary and endometrium that are independent of ovulation [104]. These changes lead to reduced oocyte quality and altered endometrial competence.

Despite ovulation, women with PCOS may experience excess E2 exposure due to a short luteal phase or subtle P4 resistance in endometrial tissues. The resulting imbalance of E2 and P4 can disrupt cell regulatory mechanisms and lead to continued E2 stimulation and P4 resistance [148]. Progesterone resistance refers to the reduced ability of the endometrial lining to respond to P4 and occurs in both ovulatory and anovulatory PCOS, although it may be more pronounced when ovulation is absent [180]. Mechanisms underlying P4 resistance include altered P4 receptor expression and/or ratios; receptor gene polymorphisms and epigenetic changes; disrupted co-regulators and microRNA; IR; and chronic inflammation [181]. E2 activates Wnt/β-catenin signaling and induces epithelial cell proliferation in the proliferative phase of the menstrual cycle, and then elevated P4 levels suppress proliferation during the secretory phase [181,182]. Normally, progesterone acts via progesterone receptors (PR-A and PR-B) to suppress proliferation, drive decidualization, and reduce inflammation [148,183]. When P4 resistance occurs, these processes become dysregulated, tipping the balance toward E2-driven growth and a proinflammatory state.

The endometrial transcriptome of ovulatory obese PCOS women was found to be significantly different from that of normal weight subjects in ovulatory menstrual cycles [184]. PCOS women had 610 differentially expressed genes, with biological processes related to inflammation (TNFR1), insulin signaling (PI3K/AKT), fatty acid metabolism, and lipotoxicity being most prominent. The most important differences in PCOS endometrium were, therefore, related to inflammation and metabolism [184].

#### 4.5.2. Endometrial Changes in Anovulatory PCOS

In chronic anovulatory PCOS, the endometrium is continuously exposed to E2 without the antiproliferative and differentiating effects of P4. This imbalance leads to altered expression of P4 receptor isoform ratios (PR-A/PR-B) and dysregulated ER-α levels, impairing endometrial decidualization and perpetuating unopposed proliferative signals. In addition, women with anovulatory PCOS have differences in gene expression in the mid-secretory endometrium—compared to controls with normal endometrium—that demonstrate P4 resistance, further exacerbating E2/P4 imbalance [185]. As a result, the combination of sustained low E2 and P4, elevated androgens and anti-Mullerian hormone, low-grade CSI, hyperinsulinemia, and IR collectively impairs decidualization and contributes to AUB, implantation failure, reduced fertility, increased risk of pregnancy complications, and postmenopausal hyperplasia (see Table 1) [10,137].

Normal endometrial decidualization relies on progesterone-driven upregulation of homeobox transcription factors such as HOXA10 and HOXA11 [183,186]. In PCOS, hyperandrogenism directly represses HOXA10, while low P4 and PR resistance further blunt stromal differentiation [187]. Concurrently, endometrial levels of leukemia inhibitory factor and downstream signal transducer and activator of transcription 3 phosphorylation are reduced, compromising the decidual response and trophoblast invasion if pregnancy ensues [188]. In addition, women with anovulatory PCOS exhibit marked downregulation of integrin αvβ3 complex—which is essential for blastocyst attachment and invasion—during the implantation window [189]. Many of these signaling pathways and transcription factors have not been investigated for their role in HMB, where no cause has been identified.

In PCOS, the endometrium is the target of endocrine, immune, biochemical, and metabolic factors present in affected women [11]. Both CSI and IR appear to exacerbate the impact of hormonal imbalance on the endometrium in PCOS. Endometrial IR in PCOS is characterized by impaired glucose transporter expression and dysregulated PI3K-AKT signaling [190]. Simultaneously, the endometrial microenvironment shows elevated pro-inflammatory cytokines (e.g., IL-6, TNF-α), reduced uterine perfusion, and oxidative stress, creating an energy-deficient, hostile environment that undermines receptivity [166]. Many of these aberrations, especially within epithelial and stromal cell subclusters, partially normalize after interventions such as metformin or lifestyle modification [14,190]. Therefore, in common with many other conditions—such as PCOS, T2DM, metabolic syndrome—some cases of HMB may result from an interaction between lifestyle and environmental factors superimposed on genetic susceptibility variants (see Section 4.7) [1].

### 4.6. Reduced Vasoconstriction, Angiogenesis, and Matrix Remodeling in HMB

Women with HMB have lower levels of PG-F2α (PGF2α) receptor [169], PG imbalance (excess PGE2/PGI2 relative to thromboxane) [169,191], and higher levels of vasodilatory PGE2 [192]. They also have reduced endothelin-1 [193], altered spiral arteriole differentiation [194], and inadequate arteriolar vasoconstriction [195] in the endometrium compared to women with normal menstrual bleeding. In addition, women with HMB have excessive local fibrinolysis (increased tissue plasminogen activator and decreased plasminogen activator inhibitor-1) [32,196], imbalanced MMP and tissue inhibitors of MMP [197], and altered angiogenic and inflammatory mediators (e.g., VEGF and cytokines) [152,198]. The additive effect of these changes makes a significant contribution to the pathophysiology of HMB [76].

Glucocorticoids promote vasoconstriction and inhibit angiogenesis in vitro and in vivo and contribute to normal menstruation [199]. Cortisol is inactivated by 11βHSD1 and 11βHSD2, and women with HMB have elevated endometrial 11βHSD2 expression [200]. Inactivation of cortisol by 11βHSD2 may promote unrestrained vessel proliferation and contribute to HMB. Novel therapies for HMB may include inhibition of 11βHSD2 or glucocorticoid replacement. A placebo-controlled randomized controlled trial (RCT) showed that 1.8 mg of dexamethasone given twice daily for 5 days in the mid-luteal phase of the menstrual cycle reduced measured blood loss by 25 mL more than a placebo (95% credible interval, 1 to 49 mL) [96].

To our knowledge, there are no published studies that directly compare endometrial PGF_2_α receptor expression, fibrinolysis, MMP, VEGF mRNA, or protein levels in women with PCOS versus controls. Similarly, there are no published histologic studies that specifically examine spiral artery differentiation or arteriolar vasoconstriction in the endometrium of women with PCOS. The development of endometrial organoid models should provide an experimental model to investigate whether similar changes contribute to endometrial dysfunction, AUB, and HMB in women with and without PCOS (see Section 4.9).

### 4.7. Genetic Insights into Endometrial Changes in PCOS

Endometrial cells from women with PCOS differentially express genes related to endometrial function (e.g., steroid hormone receptors for E2, P4, and androgens) [201], endometrial receptivity (e.g., HOXA10 and IGF-binding protein 1) [202], and inflammation (IL-8, TNFα, NFκB, CCL2, CCL5, and CCL7) [202,203]. Single-cell nuclear RNA sequencing of proliferative phase endometrium reveals a PCOS-specific endometrial signature that is characterized by an increased epithelial-to-stromal cell ratio, diminished lymphoid cell populations, and widespread dysregulation of genes involved in cell adhesion (e.g., NLGN1), cytokine signaling (e.g., integrin genes), and metabolic pathways (e.g., NRCAM, CNTN1, CD44, and ITGA6) [14]. Cell-type differentially expressed genes (DEG) correlate with metabolic and endocrine features of PCOS—such as HOMA-IR and androstenedione—suggesting that IR and HA contribute to endometrial changes in PCOS [14]. Eriksson et al. compared DEG profiles before and after treatment with lifestyle and metformin in overweight and obese IR women with and without PCOS [14]. Notably, after 16 weeks of treatment with lifestyle and metformin, there was extensive recovery of disease-specific DEGs in endometrial cells from women with PCOS.

In PCOS, elevated circulating androgens have an adverse impact on decidualization, the process in which endometrial stromal cells undergo functional transformation in preparation for implantation [19,204]. WT1 is a key transcription factor that performs a gatekeeper role as a regulator of a large network of genes involved in decidualization in human endometrial stromal cells [19,205,206]. Women with PCOS have HA, increased AR in stromal cells, and reduced WT1. Genome-wide chromatin immunoprecipitation experiments reveal that AR binds to regulatory elements on DNA normally occupied by WT1, displacing WT1 from its target promoter sites [205,206]. This competitive binding disrupts WT1’s control of genes governing cell differentiation, immune response, and angiogenesis pathways critical for decidualization. This study provides mechanistic insight into how HA dysregulates decidualization, potentially leading to symptoms such as infertility and AUB, commonly associated with PCOS.

Taken together, genetic, transcriptomic, and histological analysis of endometrial biopsies reveal PCOS-specific endometrial changes that correlate with serum endocrine and metabolic profiles. These data provide evidence that PCOS-related IR and HA have an adverse impact on endometrial function. Management with lifestyle and metformin partially restored pathways that reflect treatment-related reversal of endometrial dysfunction. Importantly, these insights provide opportunities for further research into pathway-specific treatments involving extracellular matrix organization, collagen metabolism, integrin signaling, immune cell function, and lifestyle interventions.

### 4.8. Role of the Microbiome (MB) in PCOS

#### 4.8.1. Role of the Microbiome in PCOS and Endometrial Dysfunction

As previously discussed, the endometrium in PCOS is characterized by molecular changes resulting from systemic factors, such as CSI, IR, and HA, that disrupt cyclical remodeling and alter the mucosal immune system into a more proinflammatory state. The gut MB is recognized to play a significant role in the pathogenesis of PCOS [65]. The central role of gastrointestinal “dysbiosis”—or imbalance of the MB—in PCOS has been supported and expanded by many publications investigating mechanisms involved in the described pathogenic pathways [62,207]. This theory proposes that diet, environmental, and lifestyle factors are superimposed on inherited genetic susceptibility genes that together result in symptoms and biochemical and endocrine changes in PCOS [64,65]. This theory aligns with evolutionary theories of PCOS [1] and recommendations of the international guidelines for lifestyle interventions as the first line of treatment [4], and it is supported by an extensive body of research on the role of the gastrointestinal MB in many other chronic systemic diseases [208].

Nutritional and environmental factors modify the course and prognosis of PCOS by altering the composition and function of beneficial gut microbial species, inducing oxidative stress and CSI, promoting metabolic changes and IR, and disrupting hormonal balance [64,65]. Diet quality and composition have a significant impact on gut microbial physiology that, in turn, regulates molecular pathways and alters systemic physiology in diverse tissues, including the endometrium [10,14]. The dysbiosis theory of the pathogenesis of PCOS proposes that a high-saturated-fat/high-sugar, low-fiber diet causes increased gut mucosal permeability, allowing lipopolysaccharide (LPS) to traverse the gut barrier. LPS binds with LPS-binding protein, which activates toll-like receptors on innate immune cells, upregulating NFκB-mediated inflammatory cytokine production. This cascade of events promotes CSI and IR and impacts the normal physiology of systemic tissues, including the ovaries and endometrium [65]. Similar diet-induced changes are also recognized to play a part in many chronic diseases, in addition to PCOS [63].

While there is ongoing debate regarding the specific dietary recommendations for the management of PCOS, there is general agreement about the features of a high-quality healthy diet [4]. Diet index, diet composition, diet patterns, and metabolomic studies have identified a whole-foods diet containing carbohydrates (CHOs), fats, proteins, vitamins, minerals, and contingent nutrients (e.g., polyphenols)—derived from plant-based sources and free-range or pasture raised animals—that has the potential to reduce symptoms and metabolic and endocrine changes in women with PCOS [209]. This type of diet is high in fiber and low in ultraprocessed food and environmental chemicals and has a beneficial impact on the gut MB and systemic physiology.

Recent developments in environmental epigenetics provide details of the interaction between environmental factors and genetics that highlight the dynamic interaction between environmental exposures and molecular and systemic pathways [210,211]. Detailed discussions of the molecular impact of dietary and lifestyle modification in PCOS can be found in other reviews [212,213].

**Figure 2:**
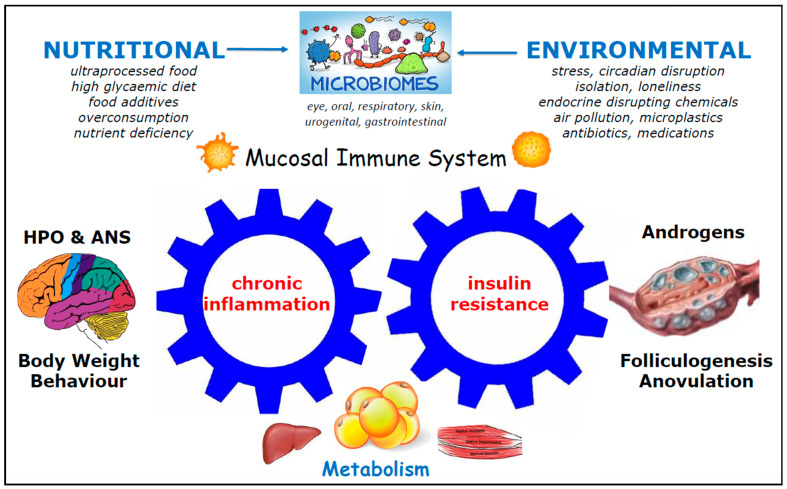
Pathogenesis of polycystic ovary syndrome. Abbreviations: HPO = hypothalamic–pituitary–ovarian; ANS = autonomic nervous system. Adapted with permission from Ref. [6]. 2023, *Life*.

Since the original publication by Tremellen and Pearce [65], further research has identified a potential role for other mucosal microbiomes, beyond the gut MB, in the pathogenesis of PCOS [175,214,215,216]. It is now clear that CSI, IR, and hormonal imbalance can be initiated at any mucosal surface via a variety of environmental risk factors [217]. A number of environmental chemicals impact the MB [218] and are absorbed, ingested, or inhaled into the human body at diverse mucosal sites, initiating CSI and metabolic and endocrine dysfunction (Figure 2) [219].

For example, nano- and microplastics enter the body via mucosal sites and can disrupt the gastrointestinal and respiratory MB [220], alter HPO function, induce oxidative stress and inflammation in reproductive tissues, and impair ovulation and fertility [221]. In addition, ingested or inhaled nanoplastics, microplastics, and microparticulate air pollution have been identified in the human endometrium and placenta, and they may contribute directly and indirectly to reproductive dysfunction and/or AUB [222,223,224]. Studies of mice have shown that long-term exposure to microplastics increases endometrial inflammatory cytokines (e.g., TNFα, IL-1β, and IL-6) and reduces fertility [224]. Human endometrial organoids exposed to microplastics show a significantly distorted shape and increased apoptosis [224]. Nano- and microplastic particles have also been found to accumulate in human endometrial stromal cells, where they induced morphological changes and cell death [223]. This is clearly concerning given the significant role of endometrial stromal cells in many aspects of normal reproductive function.

Alterations in the mucosal microbiota can result from exposure to a range of air pollutants [225]. These include particulate matter (PM 2.5, PM 5, and PM 10), polycyclic aromatic hydrocarbons, heavy metals, pesticides, gases, and many others. There has been more extensive research on ecosystem changes in the gastrointestinal and respiratory MBs, and the gut MB has been found to be particularly sensitive to diet, environmental chemicals, and medications [226]. Population-based cohort studies have provided preliminary data that microparticulate air pollution may increase the risk of PCOS [175]. Observational studies have suggested the mechanisms may include pulmonary-induced CSI [216,227], elevated fasting insulin [228], and elevated androgens [229]. A cross-sectional study of 34,832 women reported an association between irregular menstrual cycles and total suspended particulate matter in young women with PCOS diagnosed based on self-reported oligomenorrhoea and androgen excess [230].

There is a significant body of evidence linking EDCs to dysbiosis of the gastrointestinal MB, gut barrier dysfunction, CSI, metabolic disturbance and IR, endocrine imbalance, obesity, and many systemic diseases, including infertility, pregnancy complications, PCOS, and endometrial dysfunction. EDCs enter the body via air, water, and food (e.g., pesticides, herbicides, and ultraprocessed food additives). Detailed analysis is beyond the scope of the present review, and excellent reviews are available [231,232].

#### 4.8.2. Mechanistic Links Between the Gut Microbiota and Endometrial Dysfunction

The microbiome functions as an endocrine messenger that can directly regulate the HPO axis and alter ovarian and endometrial function [233]. Emerging evidence from animal studies now recognizes a reciprocal gut–gonadal communication network that contributes to reproductive health [234]. Key mediators include short-chain fatty acids (e.g., acetate and butyrate), neurotransmitters (e.g., serotonin and gamma-aminobutyric acid), inflammatory cytokines (e.g., IL-6 and TNFα), the estrabolome (a collection of gut microbial genes involved in the metabolism of E2), and alterations in the HPO axis [235]. Disruptions in intestinal microbial balance (dysbiosis) can alter ovarian function, affect oocyte development, disrupt hormone production, and reduce fertility through diverse molecular mechanisms [235]. In PCOS, for example, an imbalanced gut ecosystem augments inflammatory signaling (NF-κB) and creates a feed-forward loop of inflammation [65,236]. Lipopolysaccharide from Gram-negative bacteria can bind TLR4 on epithelial and stromal cells, activating NF-κB and escalating proinflammatory cytokine release [237,238]. This heightened inflammatory milieu can disrupt decidualization by dampening PR signaling in stromal fibroblasts [239]. In addition, randomized controlled trials have demonstrated that supplementation with probiotics [64], resistant starch [240], and synbiotics (probiotic plus prebiotic) [241] can reduce IR, drive down testosterone, and increase menstrual cycle regularity.

In summary, current evidence provides a mechanistic link between diet and environmental effects on both ovarian and endometrial dysfunction and provides the background for further investigation of the molecular mechanisms involved. It is anticipated that future research will reveal detailed molecular mechanisms regarding the role of the MB-immune network in endometrial dysfunction in PCOS. In addition, understanding the role of the distant and endometrial mucosal microbiomes may lead to novel prevention and management strategies for reproductive dysfunction and AUB.

#### 4.8.3. Role of the Endometrial Microbiome in Endometrial Dysfunction

Although the vaginal MB has been extensively characterized, there is ongoing debate regarding the typical composition of the core endometrial MB [67,242]. For instance, some studies report abundant phylum Firmicutes, with Lactobacillus as the predominant genus, followed by Bacteroidetes, Proteobacteria, and Actinobacteria. Other studies have found higher proportions of Bacteroidetes or Proteobacteria and lower levels of Lactobacillus. Nevertheless, most studies confirm the protective effect of *Lactobacillus* spp., and there is ongoing research directed at characterizing the dysbiotic endometrium. Recent studies have reported differences between fertile and infertile endometrial microbiota and a possible role for the endometrial MB in other gynecological pathology (e.g., polyps, endometriosis, fibroids, cancer) [243,244]. Determining the characteristics of the endometrial MB is a growing field that should improve scientific understanding of specific bacterial and host pathways in reproductive physiology and disease, as has occurred at other mucosal immune sites.

### 4.9. PCOS Endometrium-Derived Organoids and Endometrial Dysfunction

Historically, studies of the PCOS endometrium have used two-dimensional cell cultures and animal models to provide insights into human physiology [245]. The emergence of three-dimensional organoid systems now permits the assembly of endometrial structures that self-organize and retain features of the original epithelium [17]. Human endometrial organoids can be developed from primary endometrial cells or from pluripotent stem cells induced to form endometrial stromal fibroblasts [246,247]. Endometrial organoids form gland-like units with apico-basal polarity and undergo steroid-driven differentiation in response to E2 and P4 [89]. More recent organoid co-culture models—or “assembloids”—incorporate epithelial organoids with other autologous cells (e.g., stromal, immune, and/or endothelial cells), facilitating exploration of bidirectional crosstalk between different cell-types and mechanisms of immune tolerance [248]. Patient-derived endometrial epithelial organoids (EEOs) from women with PCOS will provide a human disease-relevant system for investigating mechanisms of endometrial dysfunction. Importantly, such cultures can originate from non-invasive menstrual fluid [249], routine biopsies, or endometrial tissue from PCOS-affected women [250].

Luyckx et al. recently reported the successful establishment of an EEO model in women with PCOS. Endometrial biopsies from overweight/obese-PCOS and lean-PCOS women were compared to BMI-matched controls in both groups [250]. Morphological assessment was performed using immunostaining, and the size of the EEO was determined after 6 days of hormone exposure. Bulk RNA-sequencing was performed to determine differences in gene expression. PCOS organoids from obese and lean samples revealed increased inflammatory gene expression (opax6ncostatin M receptor, intercellular adhesion molecule 1) and decreased size (diameter) compared to BMI-matched controls in both groups. EEOs from obese-PCOS women displayed an altered response to E2 and P4, with reduced expression of receptivity-related genes (leukemia inhibitory factor and progesterone-associated endometrial protein). The addition of dihydrotestosterone did not alter the EEO transcriptome, in accordance with the minimal expression of ARs present in epithelial cells [250].

In summary, this EEO study supports previous in vivo findings showing that the PCOS endometrium has an aberrant inflammatory gene expression and an altered response to sex hormones [185,251]. Notably, there was no difference in inflammatory gene profile or EEO size between obese- and lean-PCOS EEO. The authors hypothesized that the reduced size of both PCOS EEOs may be due to the increased inflammatory nature and/or altered mitochondrial function, as there were no differences in the levels of apoptosis markers (CC3), apoptosis gene expression, or cellular proliferation. Given the low expression of ARs in endometrial epithelial cells, coupled with their high level in stromal cells, the authors recommended that future organoid models use a co-culture system containing both cell types to investigate the effects of a hyperandrogenic environment.

### 4.10. Impact of Lifestyle Changes on Endometrial Dysfunction Where No Cause Is Identified

Modifiable risk factors—including diet, exercise, and other lifestyle factors—have been demonstrated to have a significant impact on the symptoms and biochemical and endocrine changes in PCOS [4,252]. As a result, the international evidence-based guidelines recommend lifestyle modification as the first line of management for PCOS [4]. Similarly, most chronic health conditions have modifiable risk factors that can be addressed for prevention and treatment of symptoms and underlying pathophysiology [253]. Therefore, it is important to consider the role of lifestyle modification in the management of AUB in PCOS and otherwise unexplained HMB (AUB-E).

Most of the research on AUB in PCOS has been focused on restoring cycle regularity to improve cycle control and fertility. Abundant evidence demonstrates that irregular menstruation can be improved—and regular menstruation restored—following diet and exercise interventions, as outlined in the international guidelines and many other publications [4,254]. As discussed in previous sections, there has been very little attention directed at the prevalence, molecular causes, and treatment of HMB in PCOS. It is likely that the symptom of HMB is also improved following lifestyle interventions that restore cycle regularity, but this requires further investigation in appropriately designed studies.

For example, an RCT that compared a low-glycemic index (GI) diet to a conventional healthy diet showed a significant improvement in menstrual cyclicity with a low-GI diet in women with PCOS (95% versus 63%; *p* = 0.03), but it did not report data on HMB [255]. A 2013 systematic review of diet composition studies on anthropomorphic, metabolic, psychological, and reproductive outcomes in PCOS reported that there were no differences for most outcomes between different diets [256]. The investigators concluded that a diet that reduced weight was beneficial—regardless of composition—with greater improvement in menstrual cycle regularity with a low-GI diet and greater reductions in IR for a low-GI or low-CHO diet. Prevalence and changes in HMB were not assessed. To date, no controlled dietary intervention has specifically quantified changes in menstrual blood volume loss following insulin-sensitizing low-GI or low-CHO diets.

Dietary recommendations for the treatment of IR and T2DM have changed significantly over time, with a recent resurgence of the recommendation for a CHO-restricted diet (CRD) [257]. Low-CHO diets have been conclusively shown to achieve T2DM remission in ten meta-analyses [258]. The American Diabetes Association, American Heart Association, Diabetes United Kingdom, European Association for the Study of Diabetes, and Diabetes Australia all recommend a CHO-restricted diet (CRD) as a treatment option for prediabetes and T2DM [259,260]. A CRD is also recommended by the Society of Metabolic Health Practitioners for the treatment of obesity, hypertension, metabolic syndrome, metabolic-associated steatotic liver disease, cardiovascular disease, and T2DM [261]. A CRD is a central component of the dietary recommendations for PCOS, although no specific diet is advised. Diet, environmental exposures, and other lifestyle recommendations for the treatment of AUB and HMB are completely lacking in current guidelines and should be addressed as a matter of priority.

## 5. Discussion

The present review has summarized the current understanding of normal endometrial function and regulation in reproductive-age women. This provides a background for investigating the impact of systemic IR, CSI, and hormonal imbalance on endometrial dysfunction in women with PCOS. We have identified novel mechanistic research that lays the groundwork for targeted investigations and personalized interventions. This preliminary data comes from the application of sophisticated new technological tools and models that will guide the next generation of basic, translational, and clinical research.

### 5.1. Mechanistic Implications of Novel Findings Identified in This Review

Single-cell nuclear RNA sequencing reveals how IR and HA reshape discrete endometrial cellular subpopulations, influencing inflammation and altering receptivity markers (Section 4.7). Hyperinsulinemia markedly upregulates the master transcription factor PAX6, which in turn represses cell-cycle inhibitor p27. This cascade provides a direct molecular mechanism for insulin-driven epithelial proliferation and hyperplasia in the PCOS endometrium. Hyperinsulinemia has also been shown to aberrantly activate the PI3K/AKT-NR4A1 signaling pathway in human endometrial stromal cells and disrupt decidualization (Section 4.3.2). This preliminary data offers new insights that may link hyperinsulinemia to implantation failure and endometrial-related infertility in PCOS. By targeting this pathway, we can directly address specific molecular anomalies that undermine proper decidualization in PCOS, paving the way for an approach to infertility treatment that is fine-tuned to the patient’s unique molecular profile.

Genome-wide chromatin immunoprecipitation experiments have demonstrated that elevated AR levels in PCOS competitively bind WT1 regulatory elements, displacing WT1 from its target genes. This mechanistic link explains how HA may directly impair the WT1-controlled decidualization network in stromal cells, potentially leading to defective stromal differentiation and implantation failure (Section 4.7). Patient-derived PCOS endometrial organoids have shown increased inflammatory gene expression and blunted response to E2 and P4 (Section 4.9). This human-relevant 3D model recapitulates in vivo PCOS-associated molecular defects and offers a new platform to test targeted therapies.

Together, these novel findings move beyond observational associations to identify precise molecular circuits through which IR, hyperinsulinemia, HA, and inflammation converge on endometrial cells. These mechanistic insights also have broader relevance for female reproductive health beyond PCOS. Disrupted metabolic–hormonal crosstalk underpins many uterine pathologies, such as AUB, HMB, infertility, pregnancy complications, endometrial hyperplasia, and cancer. In addition, the identification of these molecular mechanisms reflects the potential role of the endometrium as a barometer of systemic health. In the future, women with PCOS, T2DM, metabolic syndrome, inflammatory diseases, or environmental exposures could be evaluated for endometrial risk prior to the emergence of clinical reproductive problems. This has the potential to herald a new era of diagnostics and therapeutics tailored not just to PCOS, but to any condition in which metabolism and hormones intersect to compromise uterine health.

### 5.2. Current Gaps in the Molecular Understanding of Endometrial Dysfunction in PCOS

Our current knowledge outlines key pathways involving insulin, androgen, and inflammatory signaling, yet pivotal mechanisms remain unresolved. There is a lack of single-cell and special transcriptomic maps that reveal how cell location influences function across the menstrual cycle in PCOS versus controls. It is also unclear how epithelial, stromal, immune, and vascular cells interact at a molecular level. There is limited data on endometrial stem/progenitor cell function and lineage dynamics in PCOS. There is an absence of coordinated epigenomic, transcriptomic, proteomic, and metabolomic profiling in the same patient samples. There is a poor understanding of how epigenetic regulatory processes (DNA methylation, histone modification, and non-coding RNAs) interact with key signaling nodes (PI3K/AKT and Wnt/β-catenin).

Mechanistic details are missing on how hyperinsulinemia, HA, and P4 resistance integrate at the receptor and co-regulator levels in target endometrial cells. There is limited data on local intracrine steroid metabolism dysregulation (e.g., 11βHSD and aromatase) and their impact on hormone gradients in the PCOS endometrium. The characterization of the endometrial microbiome and its role in modulating immune signaling remains less advanced compared to other mucosal sites. There are gaps in defining how microbial metabolites (short-chain fatty acids and LPS) modulate endometrial immune cell function and inflammasome activation. There is also limited knowledge of exosomal and cytokine-mediated crosstalk between gut, vaginal, and endometrial microbiotas.

So far, organoid and assembloids systems have largely focused on the epithelium. Co-culture experiments with stromal, immune, and endometrial cells are needed to recapitulate in vivo complexity. Few studies have translated organoid or animal findings to human tissues or clinical biomarkers. There are almost no longitudinal studies that sample endometrial tissue across successive cycles, in the same PCOS patients, to capture trends in progression or reversibility.

### 5.3. Development of Precision Research and Targeted Therapeutic Strategies

Precision research strategies could be used to stratify PCOS endometrial biopsies by HOMA-IR (to define IR subgroup) and single-cell signatures (of PAX6/AKT activation). This would enable investigations to focus on genes regulating decidualization, immune signaling, and cell adhesion and link molecular findings to clinical outcomes (e.g., implantation failure, miscarriage, and pregnancy complications). Studies could be designed to assess the effects of lifestyle and medical interventions (e.g., metformin and GLP-1 analogs) on reversing dysfunctional changes. Patient-derived organoids could be used to screen small-molecule inhibitors of PAX6 or AKT-NR4A1 and identify potential biomarkers of responsiveness. Endometrial cytokine and COX-2 expression could be profiled across PCOS phenotypes to identify “high-inflammation” subgroups. Organoids could be used to test selective COX-2 inhibitors, anti-TNF biologics (e.g., monoclonal antibodies), or 11βHSD inhibitors for restoration of disturbed hemostatic balance in HMB and endometrial inflammation. Gut and endometrial MB analysis could be integrated to investigate the link between CSI and local inflammation. Quantification of AR and WT1 occupancy at key decidual gene promoters in endometrial samples could be used to select patients for AR-focused lifestyle and medical interventions. Co-culture organoids could be developed to test selective AR modulators (e.g., aromatase inhibitors). Tissue levels of intracrine enzymes could be measured by mass spectrometry to identify P4-resistant and E2-dominant phenotypes.

Integrated precision frameworks could be developed that combine systemic measurements (e.g., HOMA-IR, CRP, androgen levels) with endometrial single-cell/multiomics profiling to define IR-driven, CSI-driven, and hormone-driven subgroups. Biobanks could be established from patient-matched endometrial organoids to enable high-throughput screening of pathway-targeted compounds. Endometrial biomarkers (e.g., PAX6 levels, COX-2 expression, and AR-WT1 occupancy) could be used to identify trial cohorts most likely to respond to specific interventions. Multimodal treatment therapies that combine systemic agents (e.g., metformin, GLP-1 agonists, supplements), dietary-microbiome modulation (e.g., low-glycemic, high-fiber diet; probiotics; prebiotics), and/or locally delivered pathway inhibitors could be investigated for their ability to correct patient-specific molecular defects.

## 6. Limitations of the Current Narrative Review

The narrative format and broad scope of this review, while valuable for hypothesis generation and identifying novel mechanisms, introduce several constraints on translating these molecular insights directly into clinical practice. The absence of systematic inclusion criteria may introduce selection and publication bias, leading to overrepresentation of novel positive findings, while small negative studies and unpublished data remain unaddressed. The mechanistic evidence spans cell lines, animal models, and early organoid systems with variable endpoints, making it difficult to predict pathways that may be replicated in the human endometrium. There is limited validation across diverse patient samples, menstrual phases, and ethnic groups, which limits generalizability.

Current organoid models are predominantly epithelial; often cultured short-term; and do not incorporate stromal, immune, or vascular compartments needed to mirror in vivo complexity and relationships. Transcriptomic and single-cell studies predominate, whereas proteomic, metabolomic, and epigenomic data are incomplete or unavailable. There is inadequate longitudinal and interventional data that track changes in molecular defects following real-world therapies (e.g., lifestyle, medications, and supplements). Emerging environmental and microbiome-modifying factors, such as endocrine disruptors, microplastics, and microparticulate air pollution, are discussed conceptually but lack sufficient mechanistic depth or intervention trials to guide definite prevention strategies. Together, these limitations underscore the need for standardized, longitudinal, patient-matched studies and advanced co-culture organoid models to validate and refine the most promising molecular targets and interventions.

## 7. Conclusions

This review has identified the molecular pathways by which IR, CSI, and hormonal imbalance drive endometrial dysfunction and/or AUB in women with PCOS. Systemic metabolic and inflammatory changes disturb key signaling cascades in the ovary and endometrium, disrupting normal menstrual cycling and bleeding patterns, impairing receptivity and implantation, altering placentation and normal pregnancy development, and predisposing people to hyperplasia and endometrial cancer. Women presenting with unexplained irregular and/or HMB share overlapping risk factors and endometrial molecular alterations, pointing to a common underlying pathophysiology. Given the high prevalence of PCOS and AUB among reproductive-age women, these disorders represent substantial personal and public health burdens. Importantly, the pathophysiological drivers highlighted in this review—IR, CSI, and hormonal imbalance—are amenable to intervention in a large subset of patients. Prospective trials that target and reverse these metabolic and endocrine derangements should be prioritized, as they have the potential to restore normal endometrial function and improve reproductive health outcomes.

## Figures and Tables

**Table 1:** Clinical relevance and pathological mechanisms of endometrial dysfunction in PCOS.

Endometrial Problem	Pathological Findings and Mechanisms	References
Heavy menstrual bleeding	Irregular breakdown of a thickened, hyperplastic endometrium due to unopposed E2 and/or P4 deficiency, vascular fragility, and low-grade inflammation. Alterations in inflammatory mediators, hemostasis, fibrinolysis, tissue, and vascular remodeling.	[107,108]
Polyps	Chronic unopposed E2 from anovulatory cycles, together with insulin-mediated growth factors (VEGF, TGFβ-1)—and possibly HA—drive excess inflammation and focal endometrial proliferation, cystic glandular changes, stromal fibrosis, and increased vascularity.	[109,110,111,112]
Implantation failure	Defective decidualization, altered epithelium, inadequate spiral artery remodeling, immune cell imbalance, and defective extracellular matrix remodeling. Altered endometrial receptivity markers (reduced LIF, HOXA10, αvβ3 integrin, and pinopode formation) driven by HA, IR, inflammation, and obesity. Lifestyle strategies (weight loss, diet, physical activity, and circadian alignment) targeting obesity and IR play an important role.	[16,113,114]
Infertility	Combined effects of chronic anovulation and impaired endometrial receptivity reduces conception rates. Key drivers include P4 resistance, low LIF/HOXA10/αvβ3-integrin, defective pinopodes, IR, inflammation, and HA. Upregulated genes involving decidualization (HAND2, MUC1, CSF2), angiogenesis (PDGFA), and inflammation (RELA, CXCL10). Altered epigenetic expression of microRNAs. Lifestyle factors, particularly elevated BMI, are significantly associated with infertility.	[115,116]
Miscarriage	Impaired decidualization and trophoblast invasion resulting from P4 resistance, imbalanced cytokines, chronic inflammation, and metabolic dysfunction. Endometrial cells have heightened oxidative stress and dysregulated iron metabolism, leading to increased ferroptosis. Depleted antioxidant defenses (impaired glutathione peroxidase 4) compromise endometrial cell viability and placental development.	[117,118,119]
Pregnancy complications	Elevated risk of GDM, PE, FGR, PTB, and stillbirth. Placental abnormalities include defective spiral artery remodeling, spiral artery thrombosis, atherosis of basal arterioles, and failure of deep placentation, with co-existing maternal endothelial dysfunction. Underlying maternal CSI, IR, and HA, alter placental physiology and development. Lifestyle factors modify risk of pregnancy complications.	[10,28,120,121,122,123]
Hyperplasia	Prolonged estrogen exposure (unopposed by P4), obesity, decreased SHBG, dyslipidemia, elevated FAI, and IR promote abnormal growth of endometrial glands in relation to stroma +/− cytological atypia (EIN). Loss of PTEN expression, PI3K3CA mutations, and MMR deficiencies. Modifiable risks include obesity, diet, and exercise.	[107,124,125]
Endometrial Cancer	Progression from untreated atypical hyperplasia under chronic E2 stimulation, compounded by hyperinsulinemia, inflammation, and genetic mutations. Dysregulated signaling pathways (Notch, Wnt/β-catenin, PI3K/AKT/mTOR, MAPK, JAK/STAT, HER2).	[107,126,127]

Abbreviations: PCOS = polycystic ovary syndrome; E2 = 17β estradiol; P4 = progesterone; VEGF = vascular endothelial growth factor; TGFβ-1; HA = hyperandrogenism; LIF = leukemia inhibitory factor; HOXA10 = homeobox A10; IR = insulin resistance; BMI = body mass index; GDM = gestational diabetes mellitus; PE = pre-eclampsia; FGR = fetal growth restriction; PTB = preterm birth; FAI = free androgen index; CSI = chronic systemic inflammation; SHBG = sex hormone-binding globulin; EIN = endometrial intraepithelial neoplasia; PTEN = phosphatase and tensin homologue; MMR = mismatch repair; PI3K = phosphoinositide 3-kinase; AKT = serine/threonine-specific kinase; mTOR = mammalian target of rapamycin; MAPK = mitogen-activated protein kinase; JAK = Janus kinase; STAT = signal transducers and activators of transcription; HER2 = human epidermal growth factor receptor 2.

**Table 2:** Studies investigating the relationship between IR and menstrual disturbance in PCOS.

Study (Year)	Study Design Population (Country)	Key Findings	Insulin Resistance Metrics	Menstrual Cycle Length	Citation
Robinson et al. (1993)	Cross-sectional 72 PCOS 31 Controls (UK)	↓Insulin sensitivity in PCOS with oligomenorrhea cw controls (*p* < 0.01), but normal in PCOS with eumenorrhea	IV Insulin Tolerance Test	>35 days	[162]
Strowitzki et al. (2010)	Cross-sectional 118 HA PCOS (Germany)	↑HOMA-IR with amenorrhea (4.6) cw eumenorrhea (2.8) (*p* = 0.019)	HOMA-IR	>35 days	[132]
Brower et al. (2013)	Cross-sectional 494 PCOS 138 Controls (USA)	Higher mean HOMA-IR (2.2) in PCOS cw controls (1.41), after adjusting for age, BMI, and race	HOMA-IR fasting insulin	>35 days	[128]
Ezeh et al. (2021)	Cross-sectional 57 HA PCOS 57 Controls (USA)	↑Plasma glucose disappearance rate constant (kITT) in amenorrhea (1.98 +/− 0.28) cw eumenorrhea (3.33 +/− 0.51), after adjusting for age, BMI, and ethnicity	Short Insulin Tolerance Test	>35 days	[130]
Li et al. (2022)	Cross-sectional 527 PCOS 565 Controls (China)	↑HOMA-IR, ↑HOMA-β, and ↓QUICKI in women with cycles of 45–90 days cw cycles > 90 days and controls. No significant difference between cycles < 45 and 45–90 days	HOMA-IR HOMA-β QUICKI	45–90 days	[129]
Niu et al. (2023)	Retrospective 140 PCOS (China)	Dose–response relationship between ↑HOMA-IR and cycle length: eumenorrhea (1.61: CI 1.3–1.85), oligomenorrhea (2.02: CI 1.61–2.445), and amenorrhea (2.35: CI 1.96–2.75)	HOMA-IR QUICKI ISI	>35 days	[163]

Abbreviations: *↓ =* decreased; *↑* = increased; PCOS = polycystic ovary syndrome; IV = intravenous; HA = hyperandrogenism; HOMA = homeostatic model of assessment; IR = insulin resistance; QUICKI = quantitative insulin sensitivity check index; ISI = insulin sensitivity index; BMI = body mass index; kITT = plasma glucose disappearance rate constant; cw = compared with; CI = confidence interval; UK = United Kingdom; USA = United States of America.

## Data Availability

Not applicable.

## Abbreviations

AKT	Serine/threonine-specific kinase (also known as protein kinase B)
AR	Androgen receptor
AUB	Abnormal uterine bleeding
BMI	Body mass index
cAMP	Cyclic adenosine 3’5’-monophosphate
CHO	Carbohydrate
COEIN	Coagulopathy, ovulatory dysfunction, endometrial, iatrogenic, not otherwise classified
COX-2	Cyclooxygenase-2
CSI	Chronic systemic inflammation
CYP19A1	Aromatase
DEG	Differentially expressed genes
DHEA	Dehydroepiandrosterone
EEO	Endometrial epithelial organoid
ER	Estrogen receptor
E2	17β-estradiol
FIGO	International Federation of Gynecology and Obstetrics
FOXO	Forkhead box protein O
FSH	Follicle-stimulating hormone
GLUT4	Glucose transporter type 4
GnRH	Gonadotropin-releasing hormone
HA	Hyperandrogenism
HMB	Heavy menstrual bleeding
HOXA10	Homeobox 10
HPO	Hypothalamic–pituitary–ovarian
HSD	Hydroxysteroid dehydrogenase
IGF-1	Insulin-like growth factor-1
IL	Interleukin
IR	Insulin resistance
KNDy	Kisspeptin, neurokinin B, dynorphin-y
LH	Luteinizing hormone
LPS	Lipopolysaccharide
MAPK	Mitogen-activated protein kinase
MB	Microbiome
MMP	Matrix metalloproteinase
NFκB	Nuclear factor kappa-light-chain-enhancer of activated B cells
PALM	Polyp, adenomyosis, leiomyoma, malignancy, or hyperplasia
PAX6	Heterogeneous paired box 6
PCOS	Polycystic ovary syndrome
PG	Prostaglandin
PI3K	Phosphoinositide 3-kinase
PR	Progesterone receptor
P4	Progesterone
RNA	Ribonucleic acid
STAT3	Signal transducer and activator of transcription 3
TNF-α	Tumor necrosis factor-α
T2DM	Type 2 diabetes mellitus
VEGF	Vascular endothelial growth factor
WT1	Wilms tumor-1
Wnt	Wingless-related integration site
